# Surface Functionalization of Polyethersulfone Membrane with Quaternary Ammonium Salts for Contact-Active Antibacterial and Anti-Biofouling Properties

**DOI:** 10.3390/ma9050376

**Published:** 2016-05-17

**Authors:** Xiao Hu, Xiaohui Lin, Huabing Zhao, Zihao Chen, Jian Yang, Fan Li, Changjun Liu, Feng Tian

**Affiliations:** 1 The Institute of Medical Equipment, Academy of Military Medical Science, Tianjin 300161, China; huxiao0016@gmail.com (X.H.); dugechen@gmail.com (Z.C.); jianyang03@gmail.com (J.Y.); wzsliffan@gmail.com (F.L.); wzslcj@gmail.com (C.L.); 2 Physics and Chemical Department, Tianjin Center for Disease Control and Prevention, Tianjin 300170, China; linxiaohui0016@gmail.com; 3 Tianjin Key Laboratory for Prevention and Control of Occupational and Environmental Hazard, Logistics College of Chinese People’s Armed Police Forces, Tianjin 300162, China; zhaohuabing16@gmail.com

**Keywords:** quaternary ammonium salts, photografting, polyethersulfone membrane, contact-active antibacterial surface, anti-biofouling

## Abstract

Biofilm is a significant cause for membrane fouling. Antibacterial-coated surfaces can inhibit biofilm formation by killing bacteria. In this study, polyethersulfone (PES) microfiltration membrane was photografted by four antibiotic quaternary ammonium compounds (QACs) separately, which were synthesized from dimethylaminoethyl methacrylate (DMAEMA) by quaternization with butyl bromide (BB), octyl bromide (OB), dodecyl bromide (DB), or hexadecyl bromide (HB). XPS, ATR-FTIR, and SEM were used to confirm the surfaces’ composition and morphology. After modification, the pores on PES-g-DMAEMA-BB and PES-g-DMAEMA-OB were blocked, while PES-g-DMAEMA-DB and PES-g-DMAEMA-HB were retained. We supposed that DMAEMA-BB and DMAEMA-OB aggregated on the membrane surface due to the activities of intermolecular or intramolecular hydrogen bonds. Bacteria testing found the antibacterial activities of the membranes increased with the length of the substituted alkyl chain. Correspondingly, little bacteria were observed on PES-g-DMAEMA-DB and PES-g-DMAEMA-HB by SEM. The antifouling properties were investigated by filtration of a solution of *Escherichia coli*. Compared with the initial membrane, PES-g-DMAEMA-DB and PES-g-DMAEMA-HB showed excellent anti-biofouling performance with higher relative flux recovery (RFR) of 88.3% and 92.7%, respectively. Thus, surface functionalization of the PES membrane with QACs can prevent bacteria adhesion and improve the anti-biofouling activity by the contact-active antibacterial property.

## 1. Introduction

Membrane separation technology which works without the addition of chemicals and with a relatively low energy cost has a great potential value in water purification [1,2,3]. However, waterborne bacteria have a tendency to attach on the membrane surface and form a biofilm, which leads to membrane fouling and decreases the membrane performance [4,5,6].

Various methods were utilized to remove biofilm, such as biocides [7,8], mechanical cleaning [9,10,11], enzymes [12], and bacteriophage treatment [13,14]. Theoretically, the prevention is safer than removal. Membrane modification by blending, coating, or grafting was thought to be an efficient way to prevent bacteria adhesion. Zwitterionic monomers and silver nanoparticles (AgNPs) were commonly used to modify the membrane surface. Zwitterionic monomers were well-known as fouling-resistance modifiers for high hydrophily [15,16], but had no antibacterial ability. Live bacteria would still grow in water and cause fouling in a certain time. Silver nanoparticles (AgNPs) which have been shown to have excellent antibacterial properties were widely studied to coat on various materials [17,18,19]. Despite this, the dispersion and dissolution may result in loss of effectiveness, and potential toxicological impacts restrict the application of AgNPs. Contact-active antibacterial material (CAAM) is protected by covalent linkage of an antibacterial agent to a surface [20,21]. The antibacterial agent is not consumed or released and possesses advantaged in chemical stability, non-volatility, presenting long-term activity, human and environmental safety. Therefore, CAAMs are of high research and applicative value for antibacterial and antifouling materials [22,23,24,25].

Antimicrobial strategies combining polymer science have great potential in many applications. The biocidal activity could be conferred through their chemical modification [26]. Quaternary ammonium compounds (QACs) have represented one of the most visible and effective classes of disinfectants for nearly a century [27]. Via the electrostatic interactions between the cationic quaternary ammonium group and the negatively-charged bacteria cell membrane, the QAC side chain could disrupt the construction and lead to leakage of cytoplasmic material and cellular lysis. In the past decades, QACs had been covalent modified onto material surfaces by various methods and revealed to be an effective approach for rendering surfaces permanent contact-active antibacterial activity [28,29,30,31,32]. However, few papers have reported surface modification of polyethersulfone (PES) membranes with QACs to improve the antifouling activity and discussed the effect of modification to membrane flux.

Lu *et al.* [33] synthesized quaternary ammonium salt monomers with different lengths of alkyl groups from *N*,*N*-dimethylaminoethyl methacrylate (DMAEMA) and related polymers with 100% quaternization. By evaluation of their antibacterial activity, it was found that the antibacterial activities of the monomers with long alkyl chain were better than short alkyl chain. Interestingly, polymers with short alkyl chain exhibited greater antibacterial activities than their precursory monomers, but the contrary result was present in the long ones. They supposed this was due to the low solubility of the polymers with long alkyl chain in water restraining the penetration to the cell wall. Therefore, it is valuable to research the antibacterial activities of the QACs performing as contact-active agents which are not related to the dissolution.

**Figure 1:**
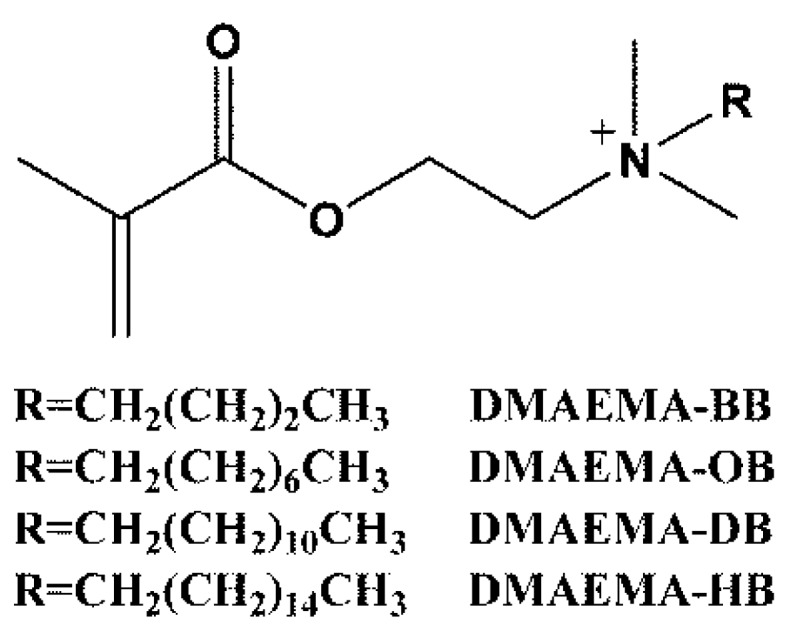
Structure of quaternary ammonium salts.

Photografting has been proved to be an effective modification of PES membranes [15,34,35]. Compared with other technologies (e.g., plasma activation [36]), it is simple, low in cost, highly selective, and can be performed at mild reaction conditions. In this research, the PES membrane was modified by antibacterial QACs through photografting. Four quaternary ammonium salts (Figure 1) were synthesized from DMAEMA and grafted on PES membranes by a UV photointiation method. The surface composition and morphology of the nascent and modified PES membranes were characterized by attenuated total reflectance spectrophotometer (ATR-FTIR), X-ray photoelectron spectroscopy (XPS), and scanning electron microscope (SEM). All PES membranes were tested with *Escherichia coli* (Gram-negative) and *Staphylococcus aureus* (Gram-positive) bacteria for the anti-bacteria and anti-adhesion performances. The anti-fouling properties were determined via filtration of *E. coli* as a model bacterial solution.

## 2. Results and Discussion

### 2.1. Surface Chemical Composition

**Figure 2:**
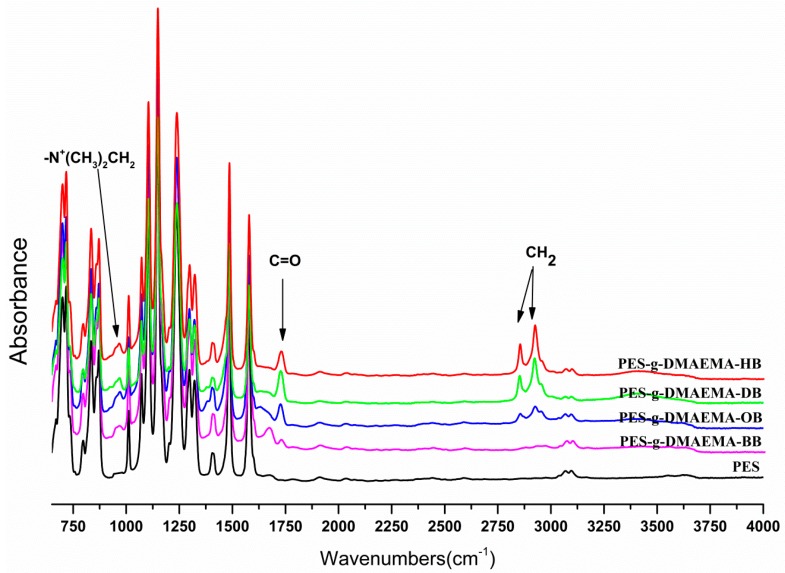
ATR-FTIR of the initial and modified PES membranes prepared with four quaternary ammonium salts monomers.

The ATR-FTIR spectra of the initial and modified PES membranes showed four quaternary ammonium salts had been grafted on membranes (see Figure 2). Compared with the initial PES membrane, a new sharp peak centered around 1728 cm^−1^ was assigned to the stretching vibration of O–C=O groups of the QACs. The other distinct peaks that appeared around 2850 cm^−1^ and 2925 cm^−1^ were attributed to the symmetric and asymmetric stretching vibrations of the –CH_2_– group. Moreover, the intensity ratio of the –CH_2_– group to O–C=O group increased with the chain length of the substituents increasing. Furthermore, an additional peak for the quaternary ammonium groups at 966 cm^−1^ was obvious in the ATR-FTIR spectra of the modified membranes.

**Figure 3:**
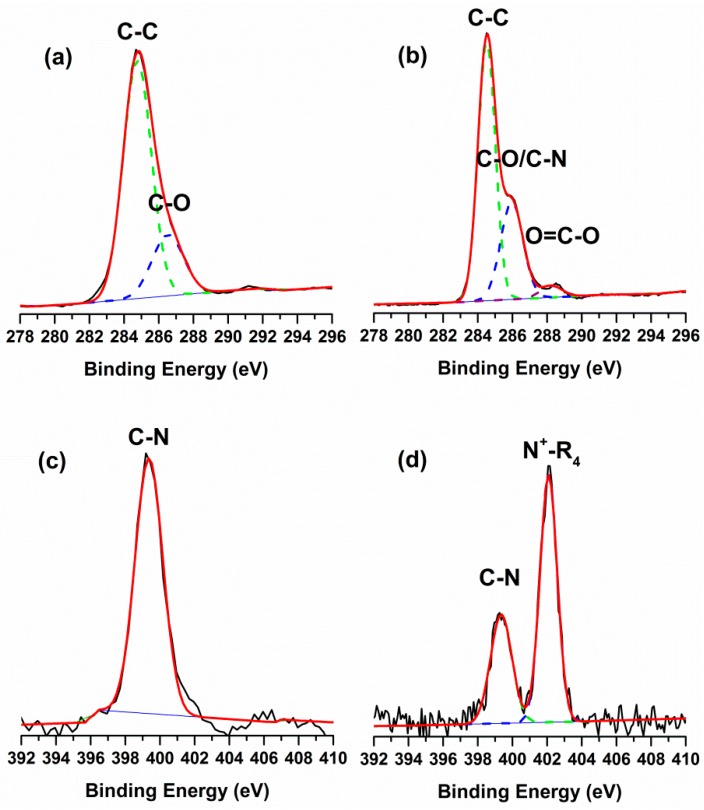
High-resolution XPS of membranes: (**a**) C 1s region of original PES; (**b**) C 1s region of PES-g-DMEAMA-HB; (**c**) N 1s region of original PE S; and (**d**) N 1s region of PES-g-DMEAMA-HB.

The surface chemical composition of the membranes was determined by XPS. The elemental analysis and grafting amount (GA) of the membranes were presented in Table 1. The initial PES membrane contained nitrogen, probably because of the additive PVP. The C/N ratio of the modified membranes was higher than the original PES membrane, as a result of higher ratio of carbon in the monomers. In addition, the C/N ratio increased along with lengthening the substituent of alkyl chain on QACs. Figure 3 showed the high-resolution C 1s and N 1s XPS spectra of the PES and PES-g-DMAEMA-HB as the representative QACs grafted membranes. The binding energies of 284.5 eV and 286.5 eV were associated with the benzene and C–O group of PES, respectively. The peak with a binding energy of 288.5 eV was associated with the O–C=O group of DMAEMA-HB. The N 1s XPS spectra of PES revealed a peak with a binding energy of 399.3 eV, which would be attributed to the additive PVP. A new peak with a binding energy of 402.1 eV which was associated with the quaternary ammonium group was detected after the grafting of DMAEMA-HB monomer onto the PES surface. These results indicated the QACs had been successfully modified on the outside surface of PES membrane.

### 2.2. Surface Morphology

**Figure 4:**
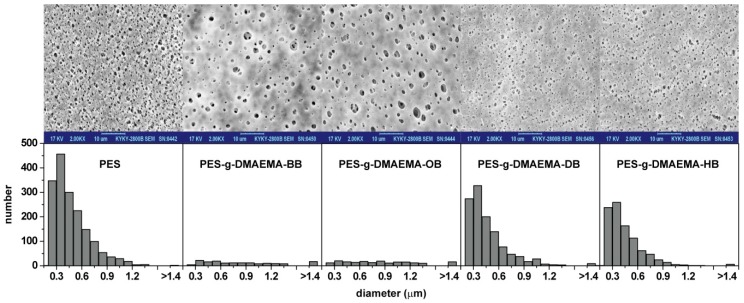
SEM images and pore size distribution of the surface of various membranes.

**Figure 5:**
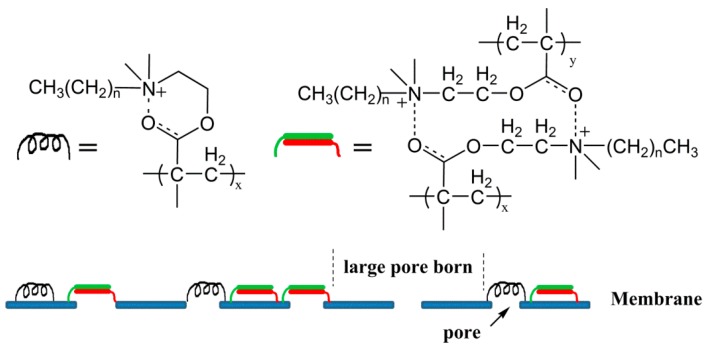
Schematic illustration for the intramolecular and intermolecular hydrogen bonds and pore blocking on the membrane surface.

The surface morphologies of various membranes were investigated by SEM, as shown in Figure 4. Compared with the initial PES membrane, the pore amounts of the modified membranes significantly reduced, because the QACs monomers or polymers covered the surface. Although PES-g-DMAEMA-BB and PES-g-DMAEMA-OB had lower GAs (193.6 μg/cm^2^ and 203.8 μg/cm^2^, respectively), the pore amounts decreased dramatically. On PES-g-DMAEMA-BB and PES-g-DMAEMA-OB surfaces, the small size pores (diameter < 1.0 μm) almost disappeared and a few large size pores bore. We suppose that the QACs have both an electro provider (C=O) and receiver (quaternary ammonium), so the hydrogen bonds could be created inter- or intramolecularly, as shown in Figure 5. This might lead the monomers or polymers assembling and blocking the pores on the membrane surface. As a small number of DMAEMA-BB and DMAEMA-OB were grafted on the membrane, the assembly could not cover the whole surface and left a skin with many large-sized pores. When the alkyl chain on the quaternary ammonium group increased (as dodecyl or hexadecyl), the steric hindrance grew up and eliminated the reaction between C=O and quaternary ammonium. As a result, the QACs were grafted equally on the surfaces. Figure 4 shows that most pores were kept on the PES-g-DMAEMA-DB and PES-g-DMAEMA-HB.

### 2.3. Antibacterial and Anti-Adhesion Activity

*S. aureus* (Gram-positive) and *E. coli* (Gram-negative) were used to assess the antibacterial ability of PES membranes grafted by QACs. The control experiment was performed using the initial PES membrane. The bacteria reductions after 24 h of incubation with various membranes were revealed in Table 2. Little change in PES group showed that bacteria were in a good condition. QACs grafted PES membranes exhibited different degree of antibacterial activities. PES-g-DMAEMA-DB and PES-g-DMAEMA-HB demonstrated strong antibacterial activities with log CFU reduction of >3 and >5, respectively. However, PES-g-DMAEMA-BB and PES-g-DMAEMA-OB were found to have weak antibacterial activity with log CFU reduction of 1.03 and 2.06 for *S. aureus*, and 1.10 and 3.90 for *E. coli*, respectively. The results were in accordance with the early reports [33]. As the alkyl chain length of the monomer on the membrane surface increased, the hydrophobic interaction between QACs and the lipid layer of the cell walls was enhanced, that resulted in stronger antibacterial activities.

**Figure 6:**
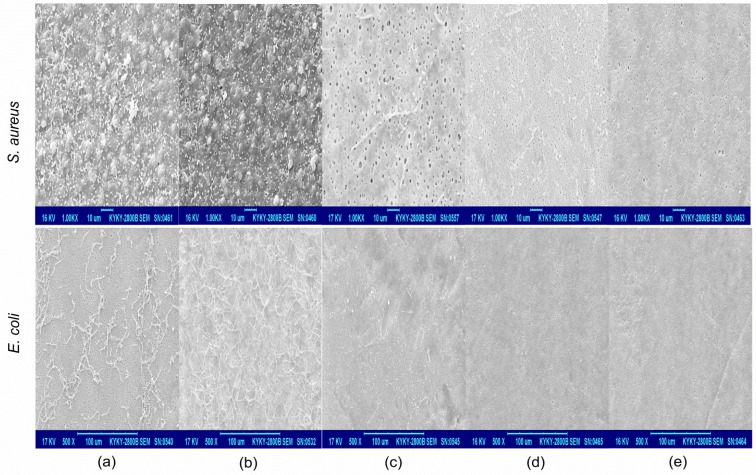
SEM images of bacteria adhesion and growth on (**a**) unmodified PES membrane; (**b**) PES-g-DMAEMA-BB; (**c**) PES-g-DMAEMA-OB; (**d**) PES-g-DMAEMA-DB; and (**e**) PES-g-DMAEMA-HB after the 24 h static adhesion experiment.

A bacteria anti-adhesion test was done for initial and modified PES membranes. The SEM images in Figure 6 showed that both of the two model bacteria (*E. coli* and *S. aureus*) could adhere to PES after 24 h of contact. It was noted that bacteria aggregated on the membrane surface and formed larger colonies which could produce many metabolites and form bio-films. As a result, the membrane surface pores were narrowed, or even blocked, and the water permeability would decrease acutely. The similar phenomenon was investigated on PES-g-DMAEMA-BB for its weak antibacterial activity. In contrast, the PES-g-DMAEMA-OB, PES-g-DMAEMA-DB, and PES-g-DMAEMA-HB had much less adhesion of bacteria on the surfaces. This might due to the stronger antibacterial activities of those membranes.

### 2.4. Anti-Fouling Performance

To evaluate the anti-fouling ability, the membranes were used to filter the *E. coli* solution as a model bacteria solution. The initial water fluxes of various membranes were illustrated in Table 3. After modified with QACs, the pure water fluxes of the modified membranes decreased because of the pore number reduction mentioned above. As a bit of pores were left on the surfaces, PES-g-DMAEMA-BB and PES-g-DMAEMA-OB retained only 34.8% and 31.0% of pure water fluxes to the original PES membrane, respectively. Therefore, the filtration experiments were not performed for them.

**Figure 7:**
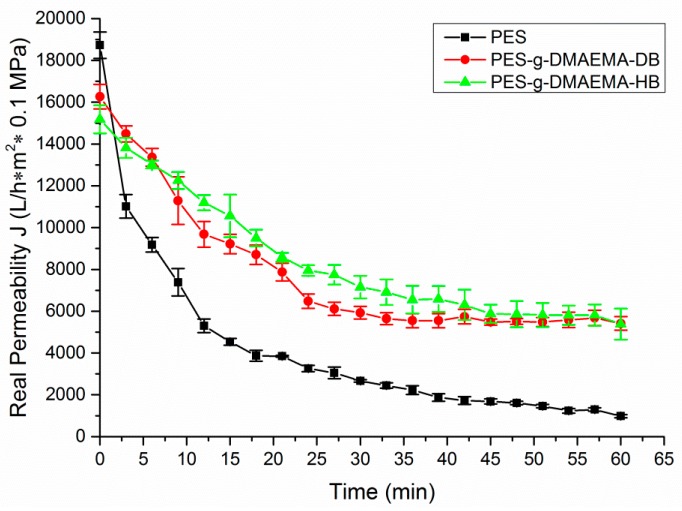
Fouling behaviors of various membranes during filtration of a bacteria solution (×10^5^ CFU/mL, *E. coli*).

Figure 7 showed the permeation fluxes of bacteria solution through PES, PES-g-DMAEMA-DB, and PES-g-DMAEMA-HB. It revealed that the permeability of nascent and modified PES membranes declined rapidly at the early stage of the filtration because of bacteria adhering on the membrane surface and blocking the pores. The similar phenomenon was observed in the previous study [37,38]. However, the water flux reductions of PES-g-DMAEMA-DB and PES-g-DMAEMA-HB were much slower and the final water fluxes were much higher (over five times) than the nascent PES membrane. The relatively steady water fluxes were observed at a later stage of filtration, which suggested that bacteria adhesion and elimination reached equilibrium on the membrane surface. These results indicated that the antibacterial layers on the membrane surface could efficiently reduce the bacteria adhesion via killing bacteria.

After the bacteria solution filtration and external cleaning, the pure water fluxes of the PES membranes were measured again. The relative flux recovery (RFR), calculated by the ratio of final pure water flux to the initial one, revealed the efficiency of cleaning bacteria. The higher RFR value means the stronger anti-adhesion ability. It was shown in Table 3 that the RFR value of PES membrane was only 55.1%, while higher values were obtained by PES-g-DMAEMA-DB and PES-g-DMAEMA-HB (88.3% and 92.7%, respectively). This suggested that bacteria attached on the membrane surface was effectively killed by DMAEMA-DB and DMAEMA-HB or lost the adhesion ability. Therefore, the modified membranes could be used for a longer time. These results were supported by the SEM images of the adhesion experiment as well.

## 3. Materials and Methods

### 3.1. Materials

Polyethersulfone (PES) microfiltration membranes were supplied by Tianjin Jinteng Laboratory Equipment Co., Ltd. (Tianjin, China). Prior to use for experiments, the membranes were washed in methanol for 1 h, then thoroughly equilibrated with deionized (DI) water. All chemical reagents were purchased by Sigma-Aldrich (St. Louis, MO, USA) and used as received. The bacteria stains were supplied by the Chinese Center for Disease Control and Prevention (Beijing, China).

### 3.2. Synthesis of Quaternary Ammonium Salts Monomers

10 mL of DMAEMA, an estimated amount of alkyl bromide [alkyl bromide/DMAEMA (1.1/1 mol)] and 30 mL dichloromethane as solvent were added in a 100 mL flask. The solution was stirred at 50 °C for 24 h. After reaction, the mixture was concentrated and dispersed in dry isopropyl ether. White needle crystals were obtained by filtering, washing with dry isopropyl ether several times and drying under vacuum at room temperature before use. The yields of QACs were 80%, 78%, 82%, 85% for DMAEMA-BB, DMAEMA-OB, DMAEMA-DB, and DMAEMA-HB, respectively.

### 3.3. Membrane Modification by Photografting

A UV system equipped with a 300 W high-pressure mercury lamp and a glass filter was used, which provided homogeneous 55 ± 5 mW/cm^2^ UV illumination (wavelength > 300 nm). Four QACs monomer solutions were prepared by dissolving in DI water and degassed by bubbling with nitrogen or ultrasonic (for DMAEMA-DB and DMAEMA-HB, as their solution would foam up by bubbling) for 30 min before use. Circular PES membranes with a diameter of 50 mm were immersed in 1% *w*/*w* monomer solutions in a Petri dish for 2 min. Then they were UV irradiated for 30 min. Thereafter, membranes were taken out, rinsed with DI water immediately and immersed in 50% ethanol/water for 2 h to remove any unreacted monomer or physically adsorbed polymer. Finally, membranes were washed with DI water again to remove ethanol and dried at room temperature for further use. The grafting amount (GA) was measured by the weight increase of modified PES membrane per area according to the following equation:

(1) $$\text{GA}\;(\mu g/{\text{cm}}^{2})=\frac{W_{1}-W_{0}}{A}$$

where *W*_0_ is the initial membrane weight (μg), *W*_1_ is the modified membrane weight (μg), and *A* is the membrane area (cm^2^).

### 3.4. Membrane Characterization

The surface chemical composition of the initial and modified PES membranes were characterized by ULVAC-PHI XPS (ULVAC, Inc., Kanagawa, Japan) and Nicolet 6700 ATR-FTIR (Thermo Fisher Scientific Inc., Waltham, MA, USA). Morphological changes of the membranes were observed by KYKY-2800B SEM (KYKY, Beijing, China) with an accelerating voltage of 6 kV. All samples were treated by gold sputtering.

### 3.5. Bacteria Tests

Gram-positive *S. aureus* and Gram-negative *E. coli* were used as model bacteria in this study. Bacteria were first cultured overnight in separate pure cultures at 37 °C with shaking at 200 rpm for 12 h in 30 mL lysogeny broth (LB) (10 g/L tryptone, 5 g/L yeast extract, and 5 g/L sodium chloride, adjusted to pH 7.0 with 1 mmol/L NaOH). After culturing, bacteria were separated from the LB by centrifugation and dispersed in phosphate-buffered saline (PBS) (pH = 7.4). The mixture went through centrifugation again to separate the bacteria from the PBS. This procedure was repeated three times to remove the nutrients. Then the resulting bacteria were then diluted with PBS to obtain a suspension of about ×10^7^ cells/mL.

Antibacterial activity was evaluated by the shaking contact tests. The membranes (d = 50 mm) were immersed into 40 mL bacteria suspension and shaken in an incubator shaker at 200 rpm and 37 °C for 24 h. After that, serial dilutions were prepared by taking 1.0 mL into 9.0 mL PBS and mixing. From these dilutions the surviving bacteria were counted by the spread plate method. Bacteria incubated with unmodified membranes were used as control. The plated dilutions were performed in triplicate and then counted.

In adhesion tests, the membranes were placed in individual wells of 24-well plate, and 100 μL of bacteria suspension was gently dropped on membranes. The bacteria adhesion was cultivated for 24 h at room temperature. Consequently, the membranes were rinsed with PBS for several times. The resulting samples were immobilized in 4.0% glutaraldehyde solution for 4 h, and then subjected to a serials of graded ethanol–water solution (10%, 20%, 30%, to 100%) and each step lasted for 20 min. Finally, samples were air-dried at room temperature for SEM observation.

### 3.6. Filtration Experiment With a Bacterial Solution

The anti-fouling ability of the initial and modified PES membranes was evaluated by a dead-end filtration system. A bacterial solution of *E. coli* about ×10^5^ cells/mL was used as the feed solution. Pure water permeability (*J*_0_) was measured until the recorded values were considered to be constant. The permeability of the bacterial solution (*J*) was measured for 60 min. Then the test solution was removed, and the membranes were washed several times with pure water. The recovered water flux was measured as *J′*. To evaluate the fouling-resistance of the membranes, the relative flux recovery (RFR, %) was calculated according to the following equations:

(2) $$\text{RFR}=\frac{J^{\prime }}{J_{0}}\times 100\%$$

## 4. Conclusions

Contact-active antibacterial material is effective to prevent biofilm formation by killing bacteria. Additionally, it can reduce bacteria amounts in the environment. That is significant to raise the efficiency of membrane separation and increase its service life. In this study, four QACs with different lengths of alkyl chains were synthesized from DMAEMA and grafted on PES membranes via UV photograft polymerization. After modification, pores on the membranes surfaces, which were grafted with short alkyl chain-substituted QACs, were narrowed and even blocked, because the hydrogen bonds might be created inter- or intramolecularly and monomers or polymers would assembled on the surfaces. For long alkyl chain-substituted QACs, the enhanced steric hindrance could eliminate the hydrogen bonds and most of the pores on the surfaces were kept. Bacteria testing revealed that the modified membranes possessed anti-bacteria ability, which increased with the carbon number of the substituted alkyl chain in monomers. It suggests that the alkyl chain length is important for the antibacterial property. Bacteria could be killed by contacting the modified membranes and few bacteria were observed on PES-g-DMAEMA-DB and PES-g-DMAEMA-HB. Biofouling experiments with a solution of *E. coli* revealed that PES-g-DMAEMA-DB and PES-g-DMAEMA-HB had lower water flux reduction and higher final water permeability than the initial PES membrane. Additionally, higher RFR values meant stronger anti-adhesion abilities of the modified membranes. Therefore, for PES membrane, UV photografting with long alkyl chain substituted QACs can be used to functionalize with contact-active antibacterial and antifouling properties.

## Figures and Tables

**Table 1:** Element composition obtained by XPS and the grafting amount of the original and modified PES membranes.

Membrane	C (%)	O (%)	N (%)	S (%)	C/N	GA (μg/cm^2^)
PES	72.20	18.01	4.89	4.70	14.76	–
PES-g-DMAEMA-BB	77.89	16.14	3.83	2.14	20.34	193.6
PES-g-DMAEMA-OB	76.88	15.99	3.49	3.64	22.05	203.8
PES-g-DMAEMA-DB	76.85	17.29	3.08	2.77	24.95	560.5
PES-g-DMAEMA-HB	78.30	16.54	2.53	2.63	30.95	835.7

**Table 2:** Reduction in viable counts of *S. aureus* and *E. coli* (×10^7^ CFU/mL) following contact with the membranes surfaces for 24 h at 37 °C.

Membrane	Log CFU/mL Reduction	
	*S. aureus*	*E. coli*
PES	0.16 ± 0.06	0.19 ± 0.05
PES-g-DMAEMA-BB	1.03 ± 0.21	1.10 ± 0.25
PES-g-DMAEMA-OB	2.06 ± 0.31	3.90 ± 0.09
PES-g-DMAEMA-DB	3.05 ± 0.39	5.40 ± 0.39
PES-g-DMAEMA-HB	3.21 ± 0.12	5.15 ± 0.09

**Table 3:** Water flux variation of different membranes.

Membrane	Initial Water Flux (*J*_0_, L/h·m^2^· 0.1 MPa)	Water Flux Reduction after Modification ^1^, %	Final Water Flux after 60 min Filtration (*J*, L/h·m^2^· 0.1 MPa)	Water Flux after Filtration External Cleaning (*J′*, L/h·m^2^· 0.1 MPa)	Relative Flux Recovery ^2^ (RFR, %)
PES	1.87 × 10^4^	–	9.80 × 10^2^	1.03 × 10^4^	55.1
PES-g-DMAEMA-BB	6.50 × 10^3^	34.8	–	–	–
PES-g-DMAEMA-OB	5.80 × 10^3^	31.0	–	–	–
PES-g-DMAEMA-DB	1.62 × 10^4^	86.6	5.41 × 10^3^	1.43 × 10^4^	88.3
PES-g-DMAEMA-HB	1.51 × 10^4^	80.7	5.38 × 10^3^	1.40 × 10^4^	92.7

^1^ The value was calculated by the initial water flux ratio of the modified membrane to PES; ^2^ The value was calculated by the ratio of *J′* to *J*_0_.

## Abbreviations

The following abbreviations are used in this manuscript:

PES	polyethersulfone
DMAEMA	dimethylaminoethyl methacrylate
QACs	quaternary ammonium compounds
CAAM	Contact-active antibacterial material
XPS	X-ray Photoelectron Spectroscopy
SEM	Electron Microscopy
ATR-FTIR	attenuated total reflectance spectrophotometer
GA	grafting amount
RFR	higher relative flux recovery
